# SPARC Regulates Transforming Growth Factor Beta Induced (TGFBI) Extracellular Matrix Deposition and Paclitaxel Response in Ovarian Cancer Cells

**DOI:** 10.1371/journal.pone.0162698

**Published:** 2016-09-13

**Authors:** David A. Tumbarello, Melissa R. Andrews, James D. Brenton

**Affiliations:** 1 Cancer Research UK Cambridge Institute, University of Cambridge, Robinson Way, Cambridge, United Kingdom; 2 University of St Andrews, School of Medicine, MBSB, North Haugh, St Andrews, United Kingdom; Fox Chase Cancer Center, UNITED STATES

## Abstract

TGFBI has been shown to sensitize ovarian cancer cells to the cytotoxic effects of paclitaxel via an integrin receptor-mediated mechanism that modulates microtubule stability. Herein, we determine that TGFBI localizes within organized fibrillar structures in mesothelial-derived ECM. We determined that suppression of SPARC expression by shRNA decreased the deposition of TGFBI in mesothelial-derived ECM, without affecting its overall protein expression or secretion. Conversely, overexpression of SPARC increased TGFBI deposition. A SPARC-YFP fusion construct expressed by the Met5a cell line co-localized with TGFBI in the cell-derived ECM. Interestingly, *in vitro* produced SPARC was capable of precipitating TGFBI from cell lysates dependent on an intact SPARC carboxy-terminus with *in vitro* binding assays verifying a direct interaction. The last 37 amino acids of SPARC were shown to be required for the TGFBI interaction while expression of a SPARC-YFP construct lacking this region (aa 1–256) did not interact and co-localize with TGFBI in the ECM. Furthermore, ovarian cancer cells have a reduced motility and decreased response to the chemotherapeutic agent paclitaxel when plated on ECM derived from mesothelial cells lacking SPARC compared to control mesothelial-derived ECM. In conclusion, SPARC regulates the fibrillar ECM deposition of TGFBI through a novel interaction, subsequently influencing cancer cell behavior.

## Introduction

The extracellular matrix (ECM) is crucial for maintaining cell homeostasis, initiating proper development of the organism, and tissue morphogenesis. During tumorigenesis, however, dysregulation of the ECM occurs which may have numerous deleterious effects on cancer progression as well as therapeutic response. Distinct tumor-host interactions and contact of the ECM with its specific paired integrin receptors can influence both therapeutic response [1–3] and tumor development [4,5]. In particular, tumors arising from ovarian cancer characteristically deposit themselves throughout the peritoneal cavity subsequently attaching to and invading mesothelial-lined tissue surfaces in an ECM-rich environment. Due to the predominant late presentation of high-grade serous (HGS) ovarian cancer, the major difficulty to successful treatment is the acquisition of drug resistance. In addition, various ECM components, including collagen VI, TGFBI, and decorin are associated with an ECM signature in ovarian cancer that has been implicated in poor prognosis and drug resistance [6–9].

We have previously shown that the secreted ECM protein transforming growth factor beta induced (TGFBI) sensitizes ovarian cancer cells to the mitotic inhibitor paclitaxel by regulating microtubule stability via integrin-mediated FAK and RhoA activation [1,3]. In addition, TGFBI has been shown to be dysregulated in a variety of cancers, including its downregulation in ovarian cancer [1,10]. Functionally, TGFBI has been shown to bind directly to a number of cell surface integrin receptors, such as αvß3, α3ß1, and α5ß1, through discrete motifs located in the conserved Fasciclin I domains and in the extreme carboxy-terminus [3,10–14]. As TGFBI interacts with multiple ECM proteins, including fibronectin and collagen, it has been proposed to act as a scaffold within the ECM coordinating distinct cellular signal transduction pathways via cell surface receptors [10]. Furthermore, *TGFBI* may act as a tumor suppressor gene, since TGFBI knockout mice develop spontaneous tumors and have upregulated cyclin D1 expression [15]. Recent identification of TGFBI’s role in chemotherapeutic response and its possible dysregulation during ovarian cancer progression led to our investigation of its organization within the extracellular compartment.

Secreted protein acidic and rich in cysteine (SPARC) was originally identified as a bone-specific protein, called osteonectin, which binds to hydroxyapatite and collagen type I [16]. SPARC is a secreted, multi-domain protein, containing an amino-terminal acidic domain that binds hydroxyapatite and calcium ions, a follistatin-like domain containing multiple cysteine residues, and a carboxy-terminal extracellular calcium-binding (EC) domain containing two EF-hand motifs. Crystal structure of the SPARC EC domain indicates that the collagen-binding site spans multiple amino acid residues within this carboxy-terminal region [17].

Functionally, SPARC has been associated with the regulation of tissue remodelling through its ability to alter matrix metalloproteinase expression [18] and modulate cell-ECM interactions via domains in both the amino- and carboxy-termini [19]. Initial studies have implicated a role in cancer progression as a result of its presence in numerous neoplastic tissues [20]. In ovarian cancer, it was suggested to have tumor suppressing properties due to its downregulation in ovarian tumors and its ability to inhibit cell growth and tumor formation in xenograft mouse models [21]. Recent data, utilizing an ovarian cancer syngeneic mouse model, suggests that the presence of host secreted SPARC limits peritoneal dissemination and ascites formation [22]. In addition, it has been shown that exogenous SPARC can promote apoptosis in ovarian cancer cells [23]. Moreover, elevated SPARC expression has been shown to occur in the activated stroma surrounding ovarian tumors [24,25], leading to the suggestion that it may be modulating the ovarian tumor microenvironment through regulation of matrix metalloproteinases, inflammation, and pro-migratory cytokines [26].

In this study, we have utilized an *in vitro* derived extracellular matrix model system to investigate the SPARC-TGFBI interrelationship. This work demonstrates SPARC as an upstream regulator of organized TGFBI fibrils in the ECM. Furthermore, our data suggests that the ability of SPARC to regulate extracellular TGFBI influences both ovarian cancer motility and response to the chemotherapeutic agent, paclitaxel.

## Materials and Methods

### Antibodies and Reagents

SPARC monoclonal antibody (clone AON-5031; 1:200 Immunofluorescence microscopy (IF), 1:1000 Western blot (WB)) was purchased from Cambridge Bioscience (Cambridge, UK). Alpha-tubulin (clone B-5-1-2; 1:3000 WB), Collagen I (clone COL-I; 1:200 IF), and Collagen IV (clone COL-94; 1:200 IF) monoclonal antibodies and actin rabbit polyclonal antibody (1:3000 WB) were purchased from Sigma-Aldrich. Purified ß1 integrin polyclonal antibody (clone Poly6004; 1:1000 WB) and anti-GST monoclonal antibody (1:3000 WB) were purchased from Biolegend (Uithoorn, Netherlands). Fibronectin (1:300 IF) and FAK monoclonal antibodies (1:1000 WB) and GFP polyclonal antibody (1:2000 WB) were purchased from Invitrogen (Paisley, UK). Affinity purified polyclonal antibody directed against TGFBI was produced by immunizing rabbits with a C-terminal peptide of human TGFBI (aa 498–683) (1:1000 WB). All antibody production was performed in collaboration with Cambridge Research Biochemicals (Cleveland, UK). TGFBI polyclonal antiserum (1:150 IF) was a kind gift from Dr. Ching Yuan (University of Minnesota, Minnesota, USA). Paclitaxel was purchased from Sigma-Aldrich, cat. no. T7402 (Dorset, UK).

### Cell culture

The ovarian cancer SKOV3 cell line and the immortalized Met5a mesothelial cell line (purchased from LGC standards, Middlesex, UK) were maintained in RPMI media supplemented with 10% (v/v) heat-inactivated FBS, 50 units/ml penicillin, and 50 μg/ml streptomycin. All other cell lines (PE01, TR175, SKOV3 TR, PEO188, and A2780) were obtained from Cancer Research UK Cell Services and cultured in appropriate growth media supplemented with 10% FBS, penicillin, and streptomycin. SKOV3 TR cells were maintained in 0.3 μM paclitaxel. Short tandem repeats genotyping was performed to confirm the identity of the cell lines. All transfections were performed with Lipofectamine 2000 (Invitrogen, Inchinnan Business Park, Paisley, UK) according to the manufacturer’s instructions. Lentivirus expressing non-target control shRNA and SPARC shRNA were purchased from Sigma-Aldrich Mission® Library. Five shRNA targets were obtained and tested for their ability to suppress protein expression assessed by Western blot. Two targets were successful and used for subsequent experiments; #2–5’-CCTGGACAATGACAAGTACAT -3’ and #5–5’-CTGCCACTTCTTTGCCACAAA-3’. Cells were infected at a MOI of 10 in the presence of 8 μg/ml of polybrene and subsequent stable pools of cells were selected in 1.25 μg/ml Puromycin.

### Vector cloning and Retrovirus production

SPARC cDNA was purchased from OriGene (Rockville, MD, USA). Full-length SPARC and SPARC lacking the carboxy-terminal 47 amino acids (aa 1–256) were PCR cloned into the HindIII and BamHI restriction sites of the pEYFPN1 vector (Invitrogen). For retrovirus production, SPARC cDNA was PCR cloned into the pLNCX2 vector (Invitrogen). Phoenix packaging cells were utilized for production of retrovirus supernatant [27]. Briefly, cells were transfected by Calcium phosphate methods (Invitrogen) according to the manufacturer’s protocol and 24 hours post-transfection, cells were refed with fresh complete growth media with viral supernatant collected three times every 18 hours. Cells were infected two times at subconfluency in the presence of 5 μg/ml polybrene and stable pools were selected in 500 μg/ml G418.

### Western blot

Cell lysates were harvested in RIPA buffer (1% Triton X-100, 10 mM Tris-HCl pH 7.4, 150 mM NaCl, 5 mM EDTA, 10 μg/ml leupeptin, and 1 mM Na_3_VO_4_). Lysates were cleared by centrifugation at 14,000xg at 4°C. Protein content was quantified by the BioRad D_c_ Protein Assay (Hertfordshire, UK). Conditioned media was harvested from cells incubated in serum-free media for 24 hours. Following the addition of 2X SDS-sample buffer, samples were boiled and loaded onto 10% SDS-PAGE gels and transferred to PVDF (Fisher Scientific UK, Leicestershire, UK). Cell-derived ECM was denuded of cells [28] and extracted directly in 2X SDS-sample buffer, boiled, and put through a 26-gauge needle before loading on SDS-PAGE. Membranes were blocked with either 5% non-fat dry milk or 3% BSA, probed with the indicated antibodies, and visualized following the addition of HRP conjugated secondary antibodies (Dako UK Ltd., Cambridgeshire, UK) and incubation with enhanced chemiluminescence (GE Healthcare UK Ltd., Buckinghamshire, UK).

### Apoptosis Assays

Cells were treated with varying concentrations of paclitaxel or DMSO vehicle control diluted in complete growth media. Following incubation at 37°C for 30 hours, both adherent and floating cells were harvested and washed once in cold PBS. The TACS Annexin-V Apoptosis kit (R&D systems Europe Ltd.) was used according to manufacturer’s instructions. For each experiment, 10,000 cell events were recorded on a BD FACS Calibur and data were analyzed with FlowJo 8.8.4 flow cytometry analysis software (Tree Star Inc., Ashland, Oregon, USA). Results were derived as the percentage of early apoptotic events (Annexin-V positive, propidium iodide negative) compared to total events. Statistical analysis was performed with GraphPad Prism® using two-way ANOVA in conjunction with Bonferroni post-hoc test.

### In vitro binding assay

For GST-binding assays, full-length SPARC, SPARC N-terminal (aa 18–134) and SPARC C-terminal (aa 154–303), SPARC aa 154–175, SPARC aa 176–256, SPARC aa 257–303, SPARC aa 154–256, SPARC aa 176–303, and SPARC aa 18–266 constructs were subcloned into the pGEX4T2 vector (GE Healthcare). All GST-fusion proteins were prepared and purified from Rosetta BL21 DE3 *E*. *coli* using Glutathione sepharose 4B beads (GE Healthcare). The pET27 TGFBI vector was a kind gift from Dr. Ching Yuan (University of Minnesota, Minnesota, USA) and recombinant TGFBI was purified from bacteria as previously described [29]. Cells were lysed in GST lysis buffer (20 mM Tris-HCl pH 7.4, 50 mM NaCl, 1% NP-40, 10% Glycerol, 0.1% ß-mercaptoethanol, and 10 μg/ml Leupeptin). Lysates were centrifuged and the supernatant was incubated with 10 μg of GST fusion protein and incubated for 2 hours at 4°C with rotation. For in vitro pull-down assays, 2 μg of rTGFBI or 2 μg human plasma fibronectin (Merck Millipore) were incubated with 10 μg of GST fusion protein. Glutathione beads were subsequently washed four times in GST lysis buffer followed by addition of 2X SDS-sample buffer and boiling. Western blot analysis was performed on samples to probe for precipitated proteins.

### Cell-derived ECM preparation and microscopy

ECM preparation from cells plated on glass coverslips or tissue culture treated dishes was carried out as previously described [28]. Briefly, glass coverslips or tissue culture treated dishes were pre-coated with 0.2% Gelatin for 1 hour at 37°C, followed by washing with PBS. Following coating, coverslips and dishes were treated with 1% glutaraldehyde for 30 minutes at room temperature, washed in PBS, and treated with 1M Ethanolamine for 30 minutes, followed by washing and storage in PBS. Cells were plated at confluency on gelatin-coated coverslips or dishes and incubated for 7–10 days in complete growth media with the addition of 50 μg/ml Ascorbic Acid and refed every 2 days. Cells were either fixed or were denuded from the matrices by incubation in PBS containing 0.5% Triton X-100 and 20 mM NH_4_OH. Matrices were washed four times in PBS and used for subsequent experiments.

For immunofluorescence microscopy, matrices were fixed in 3.7% Formaldehyde in PBS for 8 minutes. Permeabilization with 0.2% Triton X-100 in TBS was performed on intact cells when appropriate. Matrices or cells were incubated with primary antibody in TBS containing 1% BSA at 37°C for 1.5 hours, washed in TBS, incubated with either Alexa Fluor® 488 or 568 secondary antibodies (Invitrogen) in TBS containing 1% BSA, washed in TBS, and mounted in Fluorsave (Calbiochem). Images were captured on a Leica Tandem SP5 confocal microscope (Leica-Microsystems, Milton Keynes, UK) and processed with Adobe Photoshop® CS2. For quantitation of TGFBI matrix deposition, the number of enriched TGFBI immunostained foci were counted for each group from a 10X field of view and this was done for >10 fields of view and from at least 3 independent experiments. The individual data points were plotted as a box and whisker blot and represented as number of TGFBI foci/10X field of view. All statistical analysis was performed in GraphPad Prism® using one- or two-way Anova in conjunction with Bonferroni post hoc test. Quantitation of colocalisation was performed using the JACoP plugin in ImageJ and a Pearson’s coefficient was calculated between the red and green channels.

Time lapse epifluorescence video microscopy was performed using a Nikon TE2000 PFS microscope equipped with a DS-Fi1 CCD camera. SKOV3 cells stably expressing GFP were replated on Met5A-ECM derived from control shRNA or SPARC shRNA cells on ibidi 35 mm μ-dish, low (Thistle Scientific, Glasgow, UK) and images were taken using a 10X objective every 2 minutes for 15 hours using NIS elements software (Nikon Instruments Europe) in a temperature controlled and 5% CO_2_ maintained environment. Multiple fields were imaged during each experiment and at least 2 independent experiments were performed. Cell centroid tracking and analysis of motility was performed over a 10-hour period using Volocity software (PerkinElmer). All statistical analysis was performed in GraphPad Prism® using an unpaired two-tailed t-test.

## Results

### TGFBI produced by mesothelial cells forms a fibrillar matrix distinct from fibronectin

**Fig 1:**
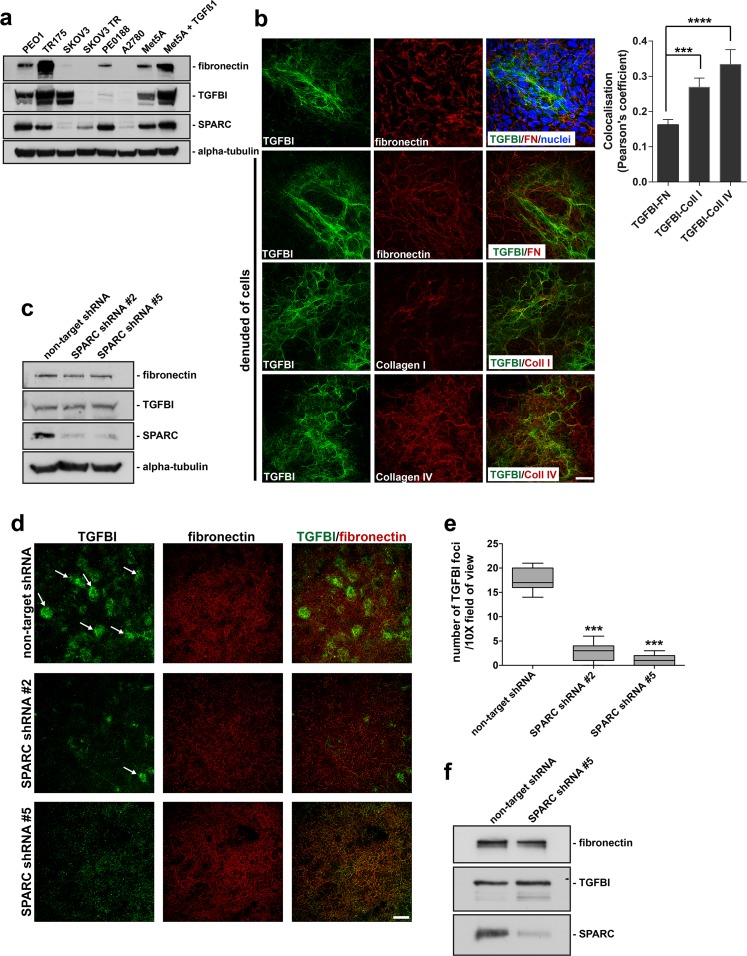
TGFBI produced by mesothelial cells forms a fibrillar matrix distinct from fibronectin and loss of SPARC expression disrupts TGFBI deposition in mesothelial-derived ECM. (a) Western blot analysis of RIPA soluble lysates derived from a panel of ovarian cancer cell lines and the Met5a mesothelial cell line either untreated or treated with TGFß1. The membrane was probed with antibodies specific to the indicated proteins. (b) Confocal microscopy of Met5a cells and extracellular matrix preparations denuded of Met5a cells cultured for 9 days, was performed following immunostaining for TGFBI, fibronectin, Collagen type I, and Collagen type IV. Hoechst dye was utilized to visualize nuclei and merged images are indicated. Scale bar 40 μm. Quantitation of colocalisation from >3 fields of view were performed and represented as a Pearson’s coefficient. Error bars indicate s.d., ****p<0.0001, ***p<0.001. (c) RIPA soluble lysates harvested from Met5a cells stably expressing either control non-target shRNA or SPARC shRNA target #2 and #5, while cultured under 3D matrix conditions. Western blot analysis was performed utilizing antibodies specific to the indicated proteins. (d) Confocal microscopy of matrix preparations from Met5a cells stably expressing non-target shRNA, SPARC shRNA #2, or SPARC shRNA #5 immunostained for TGFBI and fibronectin. Merged images are indicated. Arrows indicate individual foci. Scale bar 200 μm. (e) Quantitation of results from immunostained matrices. The number of enriched TGFBI immunostained foci were counted from each group and represented as number of TGFBI foci/10X field of view. ***represents significance from control of p<0.001, ANOVA. (f) Conditioned media was harvested from Met5a cells stably expressing either control non-target shRNA or SPARC shRNA #5, while cultured under 3D matrix conditions. Western blot was performed utilizing antibodies specific to the indicated proteins.

Ovarian cancer dissemination is enhanced by attachment within and along the peritoneal cavity, primarily lined by mesothelial cells. These cells reside in an ECM-rich environment and due to their influence on carcinogenesis [30,31], may also influence chemotherapeutic response. In addition, independently, both SPARC and TGFBI have been shown to be associated with chemotherapeutic drug resistance [1,8,32]. Moreover, the contribution of tumor- and host-derived ECM components to both ovarian cancer progression and therapeutic response remains difficult to resolve. Therefore, we assessed the relative protein expression of TGFBI and SPARC and found that both were expressed at variable levels in a panel of ovarian cancer cell lines and in the normal, immortalized Met5A mesothelial cell line both before and after transforming growth factor beta1 (TGFß1) treatment (**Fig 1A**).

In order to better understand the role of TGFBI in the ECM and how its organization may be regulated, we utilized the human immortalized Met5A mesothelial cell line to evaluate its ECM localization. TGFBI is arranged into a fibrillar matrix following a 9-day culture of the Met5A mesothelial cell line (**Fig 1B**). The cells can then be denuded from the matrix to allow better visualization of the ECM and to eliminate immunostaining derived from the cytoplasm (**Fig 1B**). Interestingly, the TGFBI fibrillar matrix is distinct from the fibrillar fibronectin matrix, indicated by very limited co-localization (**Fig 1B**). Alternatively, collagen type I in the mesothelial-derived ECM partially co-localizes with TGFBI (**Fig 1B**) and abundant amounts of collagen type IV are deposited and organized into fibrillar structures in the mesothelial-derived ECM and also partially co-localizes with TGFBI (**Fig 1B**), consistent with reports suggesting a functional interaction between TGFBI and some collagen isoforms [33].

### Loss of SPARC expression disrupts TGFBI deposition in mesothelial-derived ECM

Previous literature has suggested that SPARC may function in the ECM as a scaffold, modulating the organization of various ECM components, such as fibronectin and collagen [34,35]. Therefore, we next assessed whether SPARC was necessary for TGFBI organization in the ECM. Although ovarian cancer cells such as PEO1 express and secrete both TGFBI and SPARC (**Fig 1A**), while SKOV3 primarily only express TGFBI, their extracellular matrix is not well organized and the TGFBI fibrillar deposition is almost non-existent (**S1A Fig**). We therefore utilized the Met5A cell line since it produces a well-organized ECM with abundant amounts of deposited fibrillar TGFBI (**Fig 1A and 1B**) to evaluate the SPARC-TGFBI relationship. Stable suppression of SPARC protein expression in the Met5A cell line was achieved by introduction of two separate shRNA targets against SPARC by Lentivirus infection. Following successful suppression of SPARC expression, as assessed by Western blot and immunofluorescence microscopy (**Fig 1C; S1B and S1C Fig**), we utilized these cells to evaluate the organization of the ECM. Following culture of the stable-expressing SPARC shRNA cells for a period of 9 days, cells were denuded from the deposited matrix and the ECM was analyzed for TGFBI expression by immunofluorescence microscopy. Interestingly, in cells lacking SPARC protein expression, there was a significant decrease in TGFBI immunostaining in the mesothelial-derived matrices, although the fibronectin matrix remained unchanged (**Fig 1D**). Visualized at lower magnification, the patchy organization of TGFBI in the ECM is characterized by enriched foci of TGFBI fibrillar immunoreactivity. When quantified, the SPARC shRNA expressing cells show a significant reduction in TGFBI enriched foci within the mesothelial-derived ECM **(Fig 1E**). In contrast, the Collagen type IV matrix remained unchanged in cells stably expressing SPARC shRNA compared to non-target control shRNA (**S1D Fig**). Importantly, the loss of TGFBI immunoreactivity was not due to a decrease in overall protein expression (**Fig 1C**) or a decrease in secretion of TGFBI into the media (**Fig 1F**). Therefore, stable suppression of SPARC protein expression leads to a decrease in fibrillar TGFBI deposition within mesothelial-derived ECM.

### Modulation of SPARC expression influences TGFBI deposition in mesothelial-derived ECM

**Fig 2:**
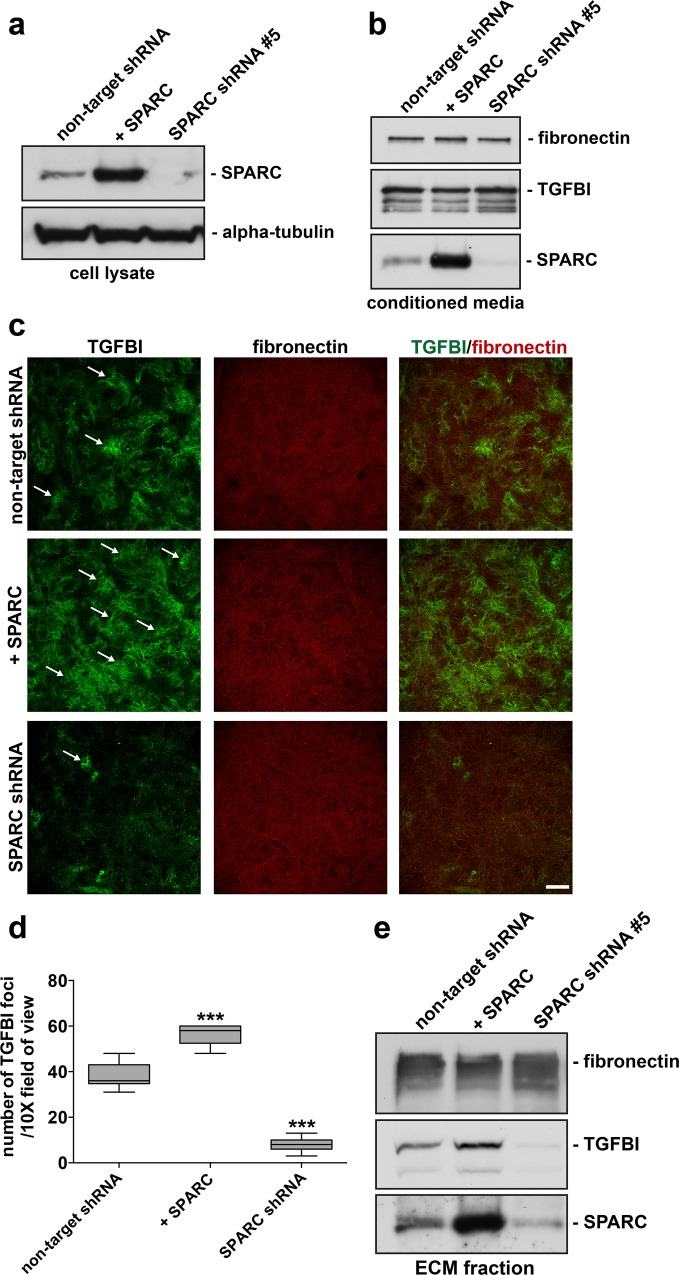
Modulation of SPARC expression influences TGFBI deposition in mesothelial-derived ECM. RIPA soluble lysates (a) and conditioned media (b) were harvested from Met5a cells stably expressing either control non-target shRNA, overexpressing SPARC cDNA, or SPARC shRNA #5, while cultured under 3D matrix conditions. Western blot analysis was performed using antibodies specific to the indicated proteins. (c) Confocal microscopy was performed on ECM preparations from Met5a cells stably expressing non-target shRNA, SPARC shRNA #5, or overexpressing SPARC cDNA immunostained for TGFBI and fibronectin as indicated. Merged images are indicated. Arrows indicate individual foci. Scale bar 200 μm. (d) Level of TGFBI immunostaining was quantified by counting TGFBI enriched immunostained foci from each group and represented by number of TGFBI foci/10X field of view. ***represents significance from control of p<0.001, ANOVA. (e) Matrix denuded of Met5a cells was solubilised in SDS-sample buffer and Western blot analysis was performed utilizing antibodies to the indicated proteins.

Our data indicates that loss of SPARC subsequently leads to a decrease in TGFBI immunostaining in mesothelial-derived ECM. We further evaluated this phenotype in the context of overexpression of SPARC in mesothelial cells. Following infection with retrovirus expressing SPARC cDNA and the generation of stable expressing pools of Met5A cells, overexpression of SPARC protein was validated by Western blot and immunofluorescence microscopy with a comparison to non-target (wild-type) and SPARC shRNA expressing cells (**Fig 2A; S1B and S1C Fig**). In contrast to a study performed in a glioblastoma cell line [36], overall expression and secretion of TGFBI was unaffected by either loss or gain of SPARC in the Met5a cell line (**Figs 1F and 2B**). Interestingly, following the 9 day culture and the production of mesothelial-derived ECM, SPARC overexpression increased TGFBI deposition in mesothelial-derived ECM, verified by quantitation of enriched TGFBI immunoreactive foci (**Fig 2C and 2D**). However, this impact on ECM deposition was not due to an overall change in Met5a cellular morphology, as paxillin-immunopositive focal adhesions remain unchanged and there was a lack of E-cadherin-mediated cell-cell junctions under all conditions (**S2A Fig**). Furthermore, extraction of the mesothelial-derived ECM from SPARC shRNA and SPARC-overexpressing cells followed by Western blot analysis verified the respective loss and gain of TGFBI deposition in the ECM fraction (**Fig 2E**). In conclusion, the level of SPARC protein expression regulates TGFBI deposition in mesothelial-derived ECM, but not the intracellular or secreted amounts of TGFBI.

### SPARC co-localizes with TGFBI in mesothelial-derived ECM

**Fig 3:**
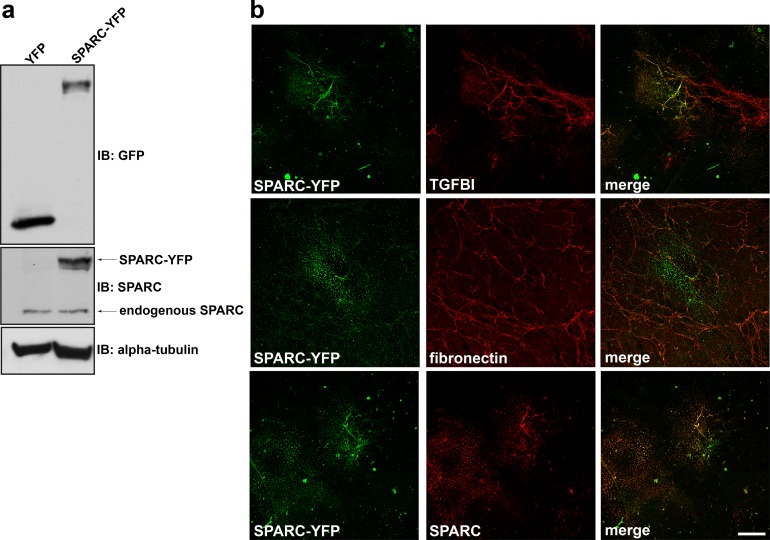
SPARC colocalizes with TGFBI in mesothelial-derived ECM. (a) Western blot analysis was performed on RIPA soluble lysates harvested from Met5a cells transfected with either YFP alone or SPARC-YFP. Immunoblotting with anti-GFP antibody recognizes YFP fusion constructs. (b) Extracellular matrix preparation was carried out from Met5a cells transfected with SPARC-YFP. Confocal microscopy was performed following immunostaining for YFP, TGFBI, fibronectin, and SPARC as indicated. Merged images are indicated. Scale bar 40 μm.

Our data indicates SPARC is a necessary component to organize fibrillar TGFBI in the ECM. We next determined whether SPARC and TGFBI co-localize in the mesothelial-derived ECM. Since antibodies against the endogenous SPARC protein revealed only weak immunostaining of SPARC (data not shown), we utilized a SPARC-YFP fusion protein to evaluate its localization following expression in Met5a cells (**Fig 3A and 3B**). Met5a cells were transiently transfected with SPARC-YFP and cultured for a period of 6 days prior to immunostaining of the cell-denuded ECM. SPARC-YFP was able to organize into an insoluble matrix characterized by punctate or fibrillar structures that was colocalized with TGFBI (**Fig 3B**). By contrast, SPARC-YFP showed minimal colocalization with fibronectin (**Fig 3B**).

### SPARC directly interacts with TGFBI via its carboxy-terminus

**Fig 4:**
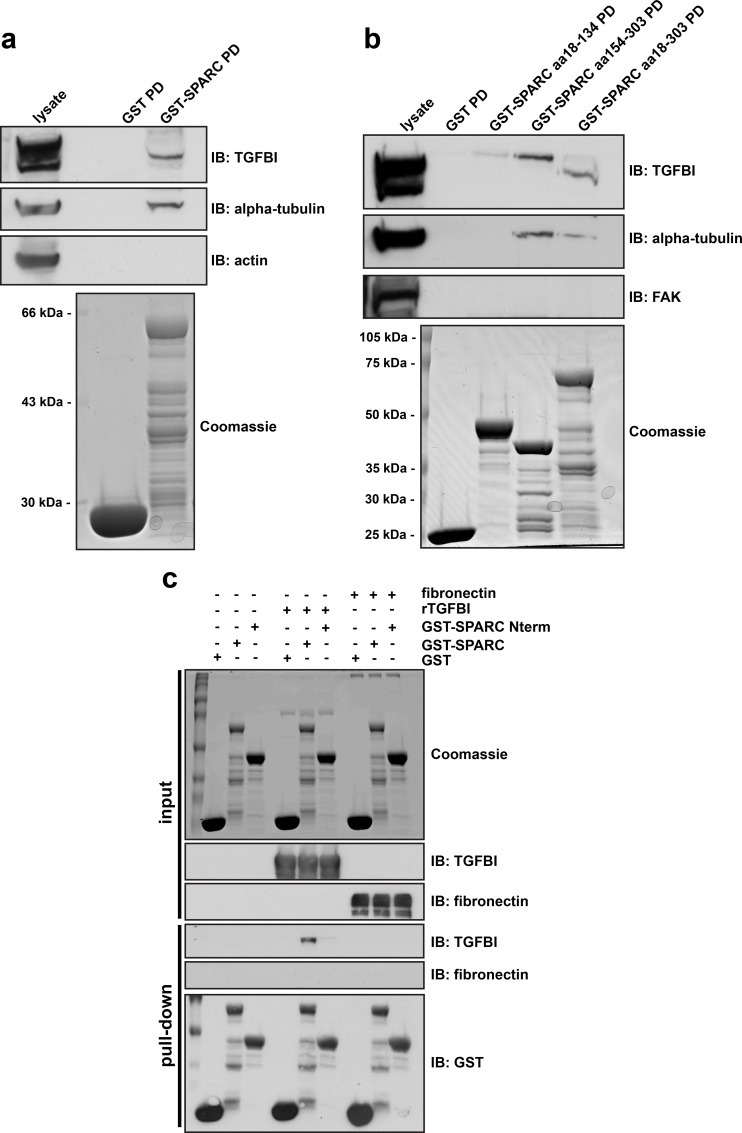
SPARC directly interacts with TGFBI via its carboxy-terminus. (a) *In vitro* GST-binding assays. Coomassie stained gel of purified GST and GST-SPARC (aa 18–303) expressed in bacteria. Western blot analysis was performed following GST pull-down from SKOV3 lysates, probed with antibodies specific to the indicated proteins. (b) Coomassie stained gel of bacterially expressed and purified GST, GST-SPARC (aa 18–303), GST-SPARC Nterm (aa 18–134), and GST-SPARC Cterm (aa 154–303) fusion proteins. Western blot analysis was performed following GST pull-down assay from SKOV3 lysates, probed with antibodies specific to the indicated proteins. (c) *In vitro* binding of purified GST-SPARC to bacterially expressed and purified recombinant TGFBI. GST, GST-SPARC (aa 18–303), or GST-SPARC Nterm (aa 18–134) fusion proteins were incubated with rTGFBI or fibronectin, followed by pull-down with Glutathione sepharose 4B beads. Subsequently, Western blot analysis was performed with antibodies specific to the indicated proteins. Coomassie stained gel represents experimental input.

Similar to TGFBI, SPARC has been reported to bind multiple ECM proteins, including different collagen isoforms, thrombospondin, and vitronectin [37,38]. Since TGFBI and SPARC have similar binding partners and co-localize in the ECM, we assessed whether TGFBI directly interacts with SPARC. First, we utilized GST-SPARC fusion proteins in pull-down assays from SKOV3 cell lysates. GST-SPARC was capable of precipitating TGFBI as well as alpha-tubulin, which was previously characterized as a SPARC binding partner [39]. The negative control, actin, was unable to bind SPARC (**Fig 4A**). As SPARC interacts with collagens via its carboxy-terminal EC domain [19,40], we determined the region of SPARC specific for its interaction with TGFBI using truncated GST-SPARC constructs and SKOV3 lysates. The carboxy-terminus of SPARC, comprising amino acids 154–303, was necessary for binding to TGFBI (**Fig 4B**).

To determine whether the interaction between TGFBI and SPARC was direct, we utilized purified recombinant TGFBI (rTGFBI) and a GST-SPARC fusion protein. Incubation of rTGFBI and GST-SPARC proteins followed by precipitation of GST-SPARC using Glutathione sepharose beads confirmed a direct interaction between the two proteins, dependent on an intact carboxy-terminus of SPARC (**Fig 4C**). Similar to work by others, SPARC was unable to interact with purified fibronectin (**Fig 4C**)[37].

### Interaction of SPARC with TGFBI is necessary for TGFBI extracellular matrix deposition

**Fig 5:**
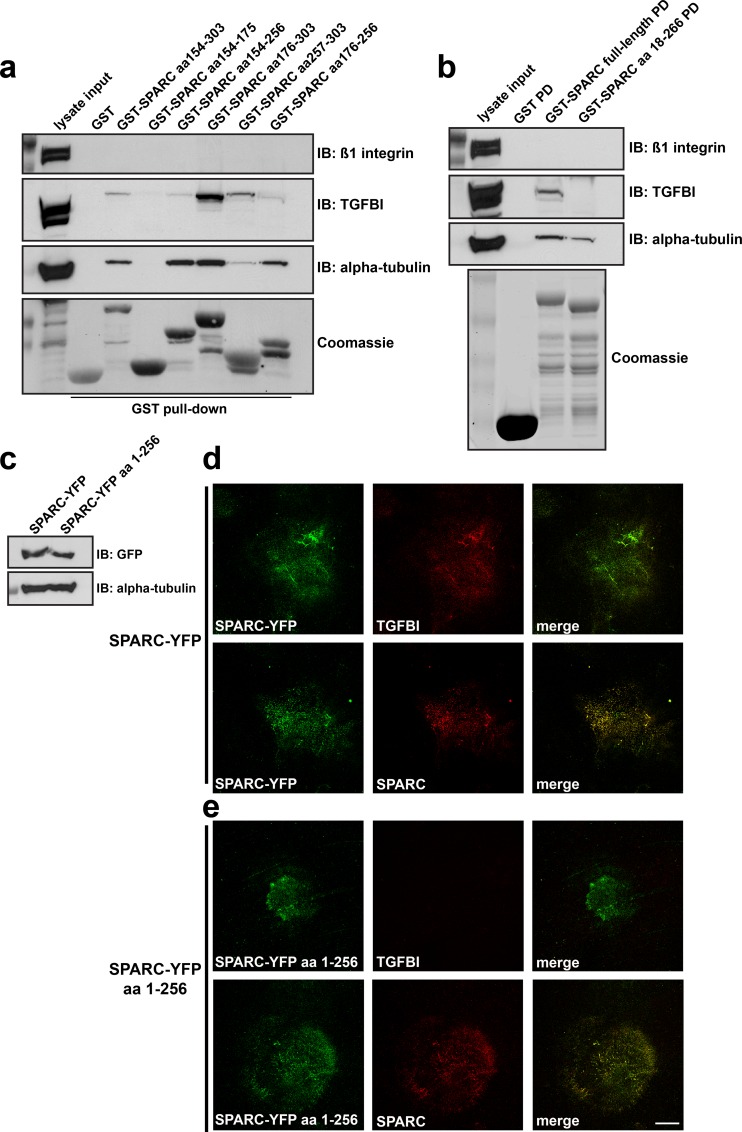
Interaction of SPARC with TGFBI is necessary for TGFBI extracellular matrix deposition. (a) GST pull-down assay from SKOV3 cell lysates utilizing truncated GST-fusion proteins derived from the Carboxy-terminus of SPARC. Following GST-pull down, Western blot analysis was performed utilizing antibodies specific to the indicated proteins. (b) GST pull-down assay from SKOV3 cell lysates using either full-length SPARC or full-length SPARC lacking the carboxy-terminal 37 amino acids (aa 18–266). Following GST-pull-down (PD), Western blot analysis was performed to the indicated proteins. Coomassie brilliant blue staining of SDS-PAGE confirms expression and purification of GST fusion proteins. (c) Full-length SPARC-YFP or SPARC-YFP lacking the carboxy-terminal 47 amino acids (SPARC-YFP aa 1–256) were transiently transfected into Met5a cells and Western blot analysis was performed to confirm their expression. (d) and (e) Extracellular matrix preparation derived from these cells following 6-day culture was subsequently processed for confocal immunofluorescence microscopy. Cell derived ECM was immunostained for YFP, TGFBI, and SPARC as indicated. Merged images are indicated. Scale bar 40 μm.

We next asked whether the TGFBI binding site on SPARC was necessary for co-localization and regulation of TGFBI deposition in the ECM. To further dissect the function of the SPARC carboxy-terminus, we performed GST pull-down assays from SKOV3 lysates with different truncated constructs derived from amino acids 154–303. The extreme C-terminus of SPARC comprising amino acid residues 257–303 was necessary to support maximum binding to TGFBI (**Fig 5A**). By contrast, alpha-tubulin predominantly bound to SPARC via a region, encompassing amino acid residues 154–256, that did not support an interaction with TGFBI (**Fig 5A**). Furthermore, a GST fusion protein encompassing full-length SPARC lacking the last 37 amino acids (a.a. 18–266) was unable to bind TGFBI, but still capable of binding alpha-tubulin (**Fig 5B**). Therefore, a region of 37 amino acids in the extreme carboxy-terminus of SPARC is necessary for its interaction with TGFBI.

We next immunostained ECM derived from Met5a cells expressing a SPARC-YFP construct encompassing amino acid residues 1–256 and therefore lacking the TGFBI binding site (**Fig 5C**). Although there was limited deposition of this construct in the ECM, there was a clear lack of TGFBI expression and co-localization compared to control cells expressing full-length SPARC-YFP (**Fig 5D and 5E**). The impaired organization of SPARC-YFP a.a. 1–256 in the ECM may be due to partial loss of the Collagen-binding site, which has been shown to be necessary to sequester SPARC in the ECM [41]. In addition, a truncated SPARC-YFP fusion protein containing amino acid residues 18–134 and therefore lacking both the Collagen and TGFBI binding site (**Fig 4**) [17], was incapable of being deposited and organized into fibrillar structures in the mesothelial-derived ECM, and did not co-localize with TGFBI (data not shown). In conclusion, SPARC and TGFBI interact biochemically, and this interaction is necessary for TGFBI deposition and their co-localization in the ECM.

### Mesothelial-derived ECM influences cancer cell motility and response to the chemotherapeutic agent paclitaxel

**Fig 6:**
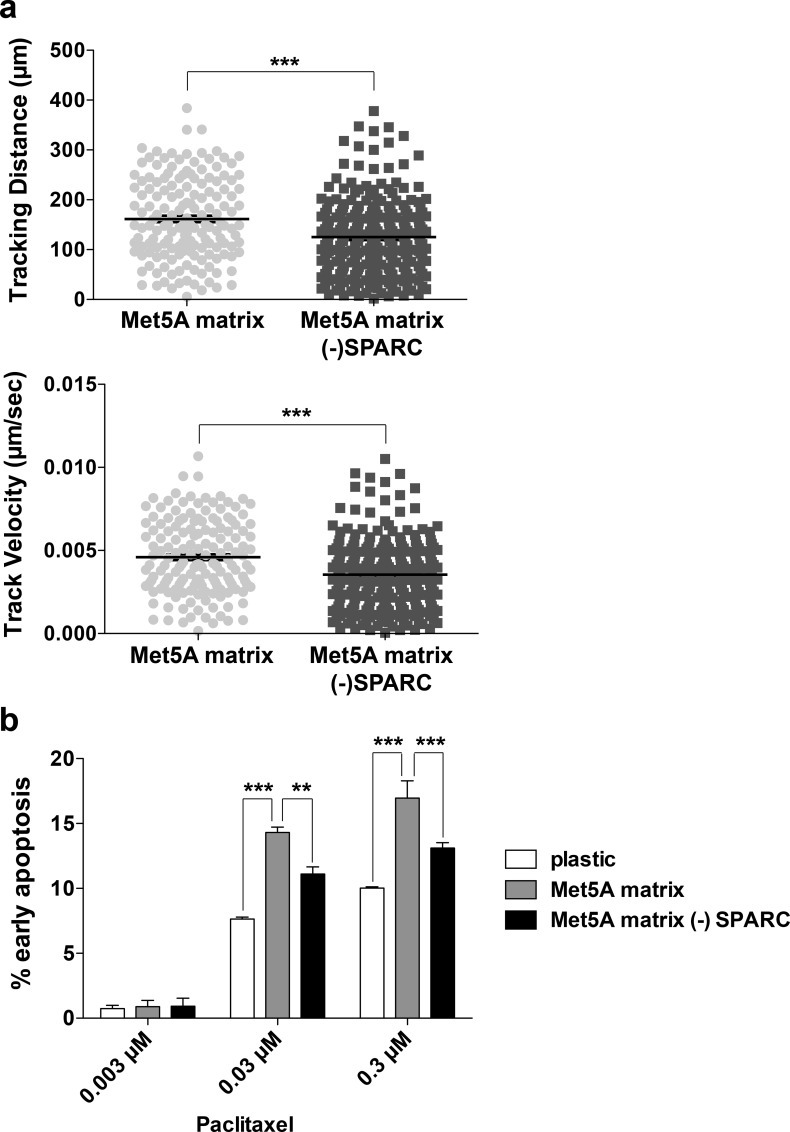
Mesothelial-derived ECM influences cancer cell motility and response to the chemotherapeutic agent paclitaxel. (a) Time lapse video microscopy was performed of SKOV3 cells plated on Met5A derived ECM derived from cells expressing either control shRNA (Met5A matrix) or SPARC shRNA (Met5A matrix—SPARC). Images were collected for 10 hours and cell centroids were tracked using Volocity software. Circles represent tracking distance and velocity of each individual cell and black bars represent the mean ±S.E.M. (b) SKOV3 cells were plated on either plastic or Met5A derived ECM derived from cells expressing either control shRNA or SPARC shRNA (- SPARC). Cells were treated with 0.003 μM, 0.03 μM, or 0.3 μM paclitaxel for 30 hours prior to staining with FITC-Annexin V and propidium iodide before analyzing by flow cytometry. Three independent experiments were performed and the results are represented by percent of cells in early apoptosis (Annexin V +, PI -). ** represents significance of p<0.01 and *** represents significance of p<0.001.

Our previous work has identified expression of TGFBI to be necessary for sensitizing ovarian cancer cells to paclitaxel-induced cell death [1]. Since SPARC regulates TGFBI deposition in the ECM, we determined how the Met5A-derived ECM could influence cancer cell motility and response to paclitaxel. First, we evaluated the motility of SKOV3 cells plated on different Met5A-derived ECMs. SKOV3 cells that stably expressed GFP were tracked, using centroid measurements, on ECM derived from Met5A cells expressing either control or SPARC shRNA. A significant decrease in tracking distance and tracking velocity was observed for SKOV3 cells plated on ECM lacking SPARC (- SPARC) compared to control matrices (**Fig 6A; S2B, S3 and S4 Figs**), while there was no change in the mode of migration as measured by the meandering index (data not shown).

We next evaluated the paclitaxel response of SKOV3 cells following plating on either plastic, control Met5A derived ECM, or Met5A SPARC shRNA derived ECM (- SPARC). Met5A-derived ECM increased the sensitivity of SKOV3 cells to paclitaxel-induced death as compared to plastic, whereas on ECM derived from SPARC shRNA cells, which lacks TGFBI, a significantly lower response to paclitaxel was observed at concentrations of 0.03μM and 0.3μM (**Fig 6B**).

## Discussion

In this study, we have defined SPARC as an upstream regulator of TGFBI deposition in the ECM. This is likely mediated by a novel interaction between TGFBI and SPARC, which occurs predominantly via the extreme carboxy-terminus of SPARC. Previously, SPARC has been shown to interact with collagen type I and is necessary for collagen fibrillogenesis in dermal fibroblasts [16,42]. Additionally, tumors derived from SPARC null mice have a more disorganized and less mature collagen matrix [43]. Furthermore, during Drosophila development SPARC is required for Collagen IV and laminin deposition in the basal laminae [44]. Our results suggest that, in a similar manner, SPARC directly interacts with and organizes TGFBI into a mature, fibrillar form within the ECM.

Previous work has suggested that SPARC regulates fibronectin organization through a direct interaction with the intracellular protein integrin-linked kinase (ILK) at the cell-ECM interface [34]. SPARC was also shown to regulate apoptosis through a direct intracellular interaction with caspase 8 [45]. Both these data suggest SPARC may be internalized from the extracellular environment in order to elicit an intracellular role [24,45], thus functioning via inside-out signalling to modulate both apoptosis and ECM organization. This is supported by data from ovarian tumor samples illustrating cancer cells lack SPARC mRNA, but do contain SPARC protein as assessed by immunohistochemistry [24]. Although our data does not support an intracellular role for SPARC with respect to modulating TGFBI deposition in the ECM, published literature suggests both a proactive intracellular and extracellular role for SPARC [24,34,35,45].

It has been suggested that SPARC may influence both tumor stroma formation, through induction of ECM expression and promote cell migration [35,46], giving further support to our findings. In addition, elevated SPARC levels correlate with increased invasiveness of ovarian cancers and poor prognosis [47,48]. Along with this, previous reports have associated SPARC expression with chemotherapy response [8,32,49]. Interestingly, it was suggested that while loss of endogenous SPARC in a mouse model of ovarian cancer increased tumor growth, it also increased the response to cisplatin chemotherapy [49]. This is supportive of data from Jazaeri et al., which indicates elevated SPARC expression in ovarian cancers that were still present following platinum-based chemotherapy [8], suggesting a role in therapeutic resistance.

Recent evidence indicates that TGFBI influences ovarian cancer response to paclitaxel by stabilizing the microtubule cytoskeleton through an integrin-dependent signalling pathway [1]. Since SPARC is secreted into the extracellular tumor microenvironment and is required for TGFBI fibrillar deposition, its expression may indirectly influence chemotherapeutic response. Interestingly, both normal and cancer cells elicit growth inhibition when exposed to extracellular-derived SPARC, but only cancer cells undergo increased apoptosis [23]. Our data illustrates that the organized mesothelial-derived ECM has the ability to sensitize ovarian cancer cells to paclitaxel-induced death. Moreover, loss of SPARC in mesothelial cell-derived ECM has a negative impact on paclitaxel-induced cancer cell death. This is in agreement with previous data evaluating the response of different cancer cell lines to taxol following plating on 3D-fibroblast derived ECM, which indicated there were a subset of cell lines that showed increased sensitivity compared to 2D culture conditions [50].

In addition, SPARC’s ability to remodel the extracellular matrix suggests it may play a role in the desmoplastic response, another potential factor influencing chemotherapeutic drug delivery and treatment response [6,35]. Thus, SPARC may have a dual role in influencing chemotherapeutic response, first, by organizing the deposition of various ECM components, thus contributing to a physical barrier to drug delivery and, second, by coordinating cellular signal transduction events through its ability to act as a molecular scaffold. This is again supported by recent evidence illustrating that in SPARC null mice ovarian tumors are more responsive to cisplatin therapy [49]. In addition, it has been suggested that SPARC secreted by macrophages increases cancer cell migration and metastasis in an integrin-dependent manner [5], consistent with our own results (Fig 6A), thus SPARC may also influence cancer dissemination in a TGFBI-dependent manner. This is supported by the work of others which suggests a correlation of SPARC and TGFBI expression with tumor metastasis [51]. Moreover, SPARC expression was previously shown to be elevated in approximately 5% of malignant epithelial cells within breast tumors and a prognostic indicator of poor outcome [52].

Our data suggests that the TGFBI binding site within the carboxy-terminus of SPARC is a relatively narrow region in comparison to the binding site for collagen, which spans multiple amino acid residues in the 5’ end of the EC domain as well as residues in the area between the two EF hands [17]. A previous report has indicated that Collagen I is necessary for retaining SPARC, as an insoluble form, in the extracellular matrix via a direct interaction [41]. Our own results do not suggest any differences in the Collagen I or the Collagen IV matrix derived from the Met5a mesothelial cell line in response to modulation of SPARC expression (S1D Fig and data not shown). However, since SPARC interacts with multiple ECM proteins [37,38], this does not rule out the possibility that modulation of SPARC expression affects other ECM components.

In conclusion, our data highlights a novel interaction between two ECM proteins, SPARC and TGFBI, both previously implicated in modulating chemotherapeutic response, whereby SPARC regulates TGFBI ECM deposition. Thus, SPARC may modulate the organization of multiple ECM components, subsequently influencing desmoplasia and drug efficacy in ovarian cancer. One caveat to consider is that since SPARC may alter both the tumor microenvironment and treatment response, therapies targeted to modulate SPARC function may affect both delivery and response to chemotherapy. In addition, the absence of SPARC may promote tumor dissemination, as was illustrated in a mouse ovarian cancer model [22], although our results would suggest this may not be due to an effect on cell migration. Regardless, SPARC is likely a key component in organizing ECM proteins such as TGFBI in the tumor microenvironment and both may be prognostic indicators of chemotherapeutic response.

## Supporting Information

S1 Fig SKOV3 and PEO1 cells have a disorganized ECM, while loss of SPARC has no effect on Met5A derived Collagen IV matrix organization. (a) Confocal microscopy of ECM derived from SKOV3 and PEO1 cultured for 9 days prior to ECM extraction. ECM was immunostained for TGFBI and fibronectin as indicated. Scale bar 40 μm. (b) and (c) Confocal microscopy of Met5A cells stably expressing either control non-target shRNA, overexpressing SPARC cDNA, or expressing SPARC shRNA following immunostaining for TGFBI and SPARC. Nuclei are visualized with Hoechst stain and merged images are shown. (d) Met5A cells stably expressing either non-target control shRNA or SPARC shRNA were cultured for a period of 9 days prior to cells being denuded from the deposited ECM. Confocal microscopy was performed following immunostaining for TGFBI and Collagen IV. Scale bar = 40 μm.
(TIF)

S2 Fig Modulation of SPARC expression has no effect on E-cadherin intracellular localisation or focal adhesion organisation, while SKOV3 cells have decreased migration on ECM derived from SPARC depleted Met5A cells. (a) Confocal microscopy of Met5A cells either stably expressing control non-target shRNA, overexpressing SPARC cDNA, or expressing SPARC shRNA following immunostaining for TGFBI, E-cadherin, and paxillin. Nuclei are visualized with Hoechst stain and merged images are indicated. Scale bar = 40 μm. (b) Migration tracks of SKOV3 cells on either Met5A-ECM derived from control shRNA cells or SPARC shRNA cells following time lapse epifluorescent microscopy. Images were collected every 2 minutes for 10 hours and processed with Volocity software.
(TIF)

S3 Fig SKOV3 cells plated on Met5A-derived ECM from control shRNA treated cells. SKOV3 cells are stably expressing GFP and plated on ECM derived from control shRNA treated Met5A cells. Images captured every 2 minutes. Time display indicates hours:minutes.
(AVI)

S4 Fig SKOV3 cells plated on Met5A-derived ECM from SPARC shRNA treated cells. SKOV3 cells are stably expressing GFP and plated on ECM derived from SPARC shRNA treated Met5A cells. Images captured every 2 minutes. Time display indicates hours:minutes.
(AVI)
