# Supplementary figures and images for: SPARC Regulates Transforming Growth Factor Beta Induced (TGFBI) Extracellular Matrix Deposition and Paclitaxel Response in Ovarian Cancer Cells
Source: PLoS One. 2016 Sep 13;11(9):e0162698. doi: 10.1371/journal.pone.0162698 (PMC5021370; doi:10.1371/journal.pone.0162698)

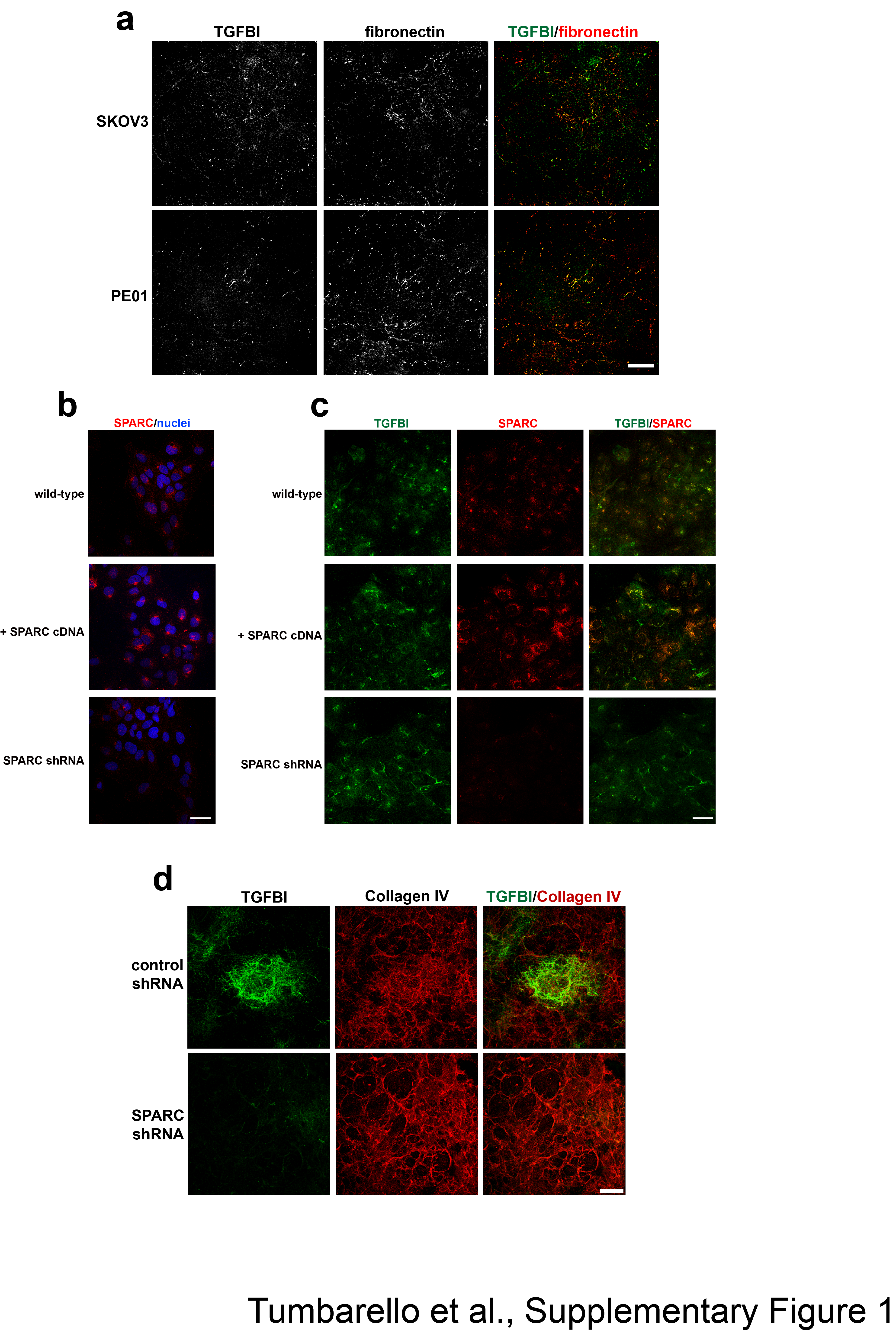

Supplement: S1 Fig — (a) Confocal microscopy of ECM derived from SKOV3 and PEO1 cultured for 9 days prior to ECM extraction. ECM was immunostained for TGFBI and fibronectin as indicated. Scale bar 40 μm. (b) and (c) Confocal microscopy of Met5A cells stably expressing either control non-target shRNA, overexpressing SPARC cDNA, or expressing SPARC shRNA following immunostaining for TGFBI and SPARC. Nuclei are visualized with Hoechst stain and merged images are shown. (d) Met5A cells stably expressing either non-target control shRNA or SPARC shRNA were cultured for a period of 9 days prior to cells being denuded from the deposited ECM. Confocal microscopy was performed following immunostaining for TGFBI and Collagen IV. Scale bar = 40 μm. (TIF) [file pone.0162698.s001.tif]

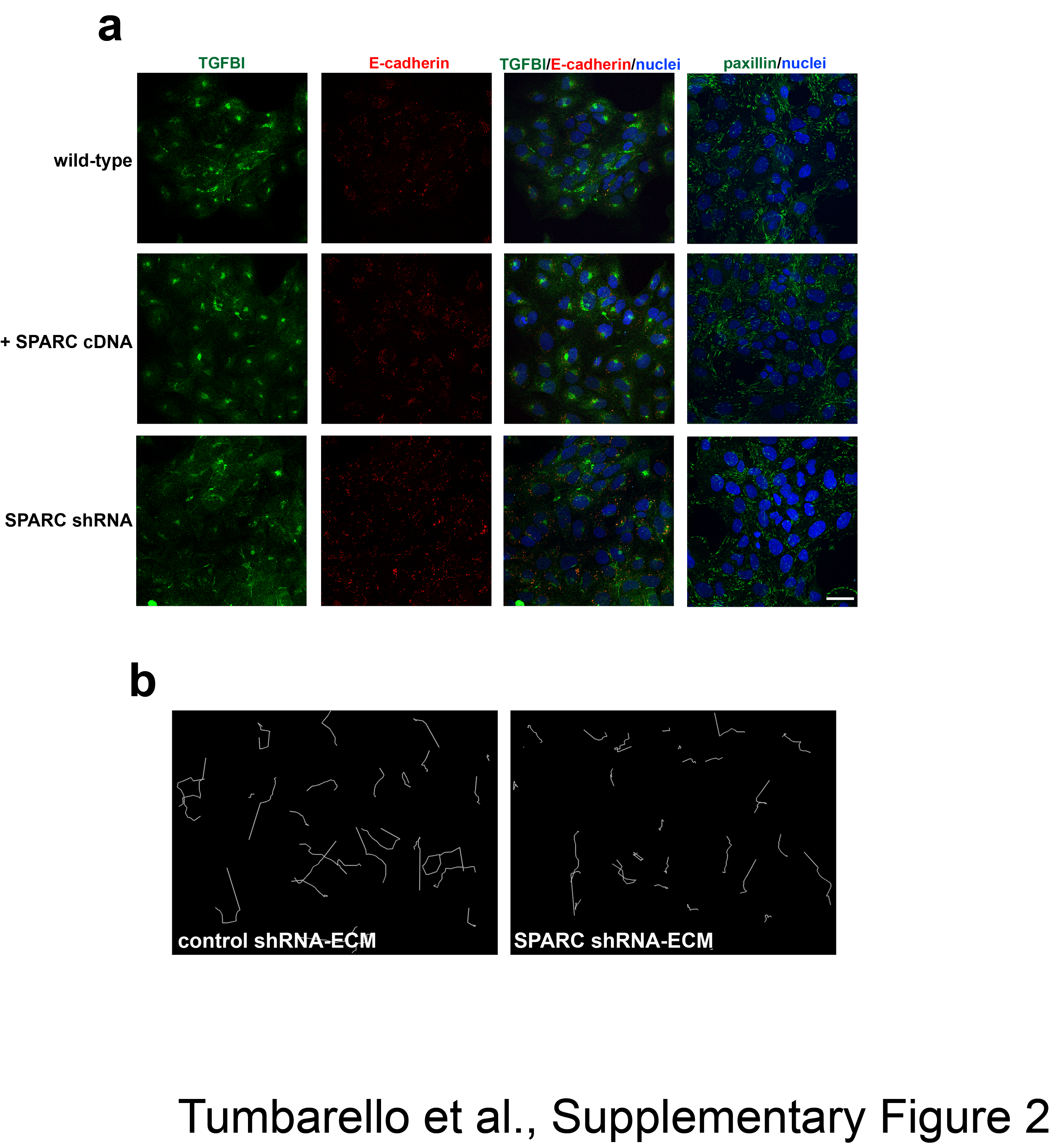

Supplement: S2 Fig — (a) Confocal microscopy of Met5A cells either stably expressing control non-target shRNA, overexpressing SPARC cDNA, or expressing SPARC shRNA following immunostaining for TGFBI, E-cadherin, and paxillin. Nuclei are visualized with Hoechst stain and merged images are indicated. Scale bar = 40 μm. (b) Migration tracks of SKOV3 cells on either Met5A-ECM derived from control shRNA cells or SPARC shRNA cells following time lapse epifluorescent microscopy. Images were collected every 2 minutes for 10 hours and processed with Volocity software. (TIF) [file pone.0162698.s002.tif]
